# Molecular Mechanisms of Cadmium-Induced Apoptosis in Fish Cells: A Review

**DOI:** 10.3390/ijms27094035

**Published:** 2026-04-30

**Authors:** Yun Dai, Yongyao Guo, Dongjie Wang, Wei Luo, Jixing Zou, Zongjun Du

**Affiliations:** 1 Fisheries College, Sichuan Agricultural University, Chengdu 611130, China; pdai0717@gmail.com (Y.D.); g353746433@gmail.com (Y.G.); dongjiewang1995@gmail.com (D.W.); lwdjn@live.cn (W.L.); 2 College of Marine Sciences, South China Agricultural University, Guangzhou 510642, China

**Keywords:** fish, cadmium exposure, apoptosis, signaling pathway, molecular mechanism

## Abstract

Cadmium (Cd) is a typical heavy metal pollutant in aquatic environments. It enters fish through the gills, digestive tract, and body surface, and accumulates mainly in the liver and kidneys, with species- and tissue-specific distribution. Cadmium triggers apoptosis by inducing oxidative stress, calcium imbalance, and DNA damage. These signals are integrated and amplified by the mitogen-activated protein kinase (MAPK), nuclear factor kappa B (NF-κB), phosphatidylinositol 3-kinase (PI3K)/AKT, and nuclear factor erythroid 2-related factor 2 (Nrf2) pathways, ultimately activating three downstream apoptotic execution pathways: the death receptor, mitochondrial, and endoplasmic reticulum stress pathways. These three pathways form an interactive network through molecular nodes such as BH3 interacting domain death agonist (Bid), Ca^2^^+^, c-Jun N-terminal kinase (JNK), and C/EBP homologous protein (CHOP), synergistically amplifying the apoptotic effect, with the mitochondrial pathway playing a central role. Cadmium-induced apoptosis is dose-dependent: low concentrations activate protective responses, whereas high concentrations strongly promote apoptosis. Current research gaps remain regarding dynamic pathway crosstalk, chronic low-dose effects, species differences, and fish-specific apoptotic molecules (e.g., caspase-12 homologs). Future studies should focus on constructing multidimensional response maps, clarifying pathway activation thresholds and interaction contributions, and developing composite protective strategies based on Nrf2 activators, metal chelators, and antioxidants, thereby promoting translation into ecological risk assessment and aquaculture pollution control.

## 1. Introduction

Cadmium (Cd) is a typical heavy metal pollutant that enters aquatic environments through rock weathering, industrial wastewater discharge, and atmospheric deposition [1,2,3,4,5]. A survey of a cadmium-polluted town in southeastern China reported that over 90% of total cadmium emissions were discharged into water bodies in 2015 [6]. In water, cadmium mainly exists as the divalent ion (Cd^2+^). Although complexes formed with anions such as Cl^−^ and SO_4_^2−^ are less toxic, free Cd^2+^ remains highly bioavailable and toxic [7,8]. Studies have shown that cadmium concentrations of 50–100 μg/L in water can induce toxic responses in fish cells [9,10,11]. Due to its chemical similarity to essential metal ions such as calcium (Ca^2+^) and zinc (Zn^2+^), Cd^2+^ enters cells through carriers including calcium channels and zinc transporters (e.g., the ZIP family). Once inside, Cd^2+^ can displace essential ions from metalloproteins, disrupting their normal functions. In addition, Cd^2+^ has a high affinity for sulfhydryl (-SH) groups in proteins, leading to structural and functional abnormalities [12,13]. These properties form the molecular basis of cadmium cytotoxicity.

Apoptosis is a genetically regulated program of cell death that plays a critical role in maintaining tissue homeostasis and eliminating damaged cells [14]. Depending on the initiating signal, apoptosis can be executed through three major pathways. The extrinsic pathway (death receptor pathway) is activated when death receptors on the cell membrane (e.g., Fas, tumor necrosis factor receptor 1 (TNFR1)) receive extracellular signals, leading to the activation of caspase-8/10 [15]. The intrinsic pathway (mitochondrial pathway) is triggered by intracellular stress (e.g., DNA damage, oxidative stress), causing the release of cytochrome c from mitochondria and subsequent activation of caspase-9 [16,17]. The endoplasmic reticulum (ER) stress pathway is activated by disturbances in ER homeostasis (e.g., disrupted calcium balance, accumulation of unfolded proteins), which activate caspase-12 or downstream signals [18]. Importantly, these three pathways are not independent; they interconnect through key molecules such as Bid, JNK, and Ca^2+^, forming a complex network that collectively determines cell fate [19,20].

Synthesis of the complete molecular mechanisms is still lacking, due to the complex chemical forms of cadmium, the diversity of fish species, and the extensive crosstalk among apoptotic pathways. Therefore, this review systematically summarizes recent research progress on cadmium-induced apoptosis in fish. It first describes the absorption, transport, and accumulation of cadmium in fish. It then analyzes the molecular mechanisms of cadmium-induced apoptosis from two levels: upstream initiating factors (oxidative stress, calcium imbalance, DNA damage) and downstream execution pathways (death receptor, mitochondrial, and ER stress pathways), with a particular focus on the crosstalk among these pathways. Finally, this review identifies current research gaps and proposes future directions, aiming to provide a theoretical reference for aquatic ecotoxicology studies, ecological risk assessment, and pollution control related to cadmium.

## 2. Absorption, Transport, and Accumulation of Cadmium in Fish

### 2.1. Cadmium Uptake by Fish

**Figure 1:**
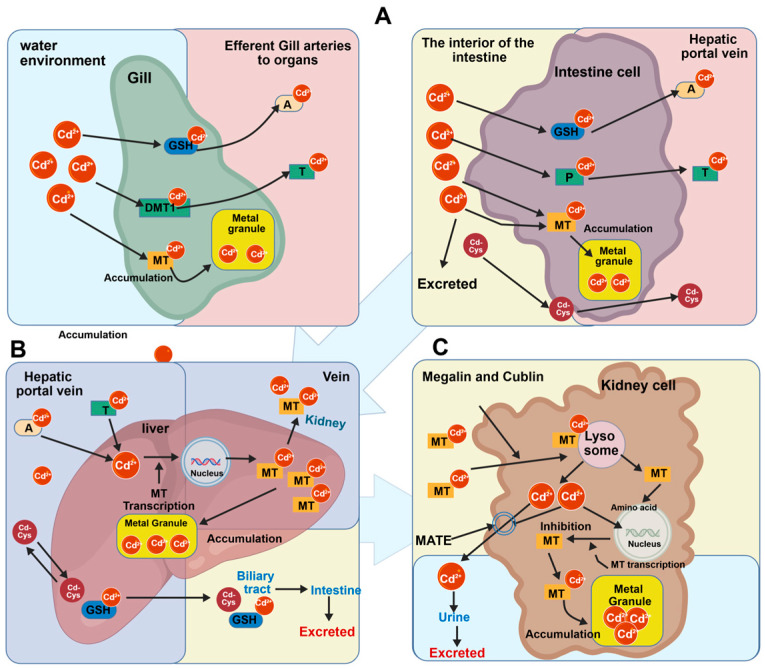
Schematic diagram of cadmium (Cd^2+^) metabolism and toxicity mechanisms in fish. (**A**) Uptake and detoxification in gills and intestine. Cd^2^^+^ enters cells and ischelated by glutathione (GSH), cysteine, or metallothionein (MT), forming metal granules for accumulation. (**B**) Hepatic metabolism and storage. Cd^2^^+^ induces MT synthesis; the resulting Cd-MT complexes are either stored as metal granules or transported to the kidney. (**C**) Renal reabsorption and excretion. Cd-MT complexes are taken up via Megalin/Cubilin, degraded in lysosomes, and released Cd^2^^+^ is excreted into urine.

Fish can absorb cadmium from water through several routes (Figure 1A). The gills are the primary site of uptake, where dissolved Cd^2+^ enters gill epithelial cells via passive diffusion or specific ion channels [21]. In some teleost fish, due to the chemical similarity between cadmium and calcium, calcium channels mediate the transmembrane transport of Cd^2+^ [22]. Additionally, zinc transporters (e.g., the ZIP family) on the gill epithelial membrane are involved in cadmium uptake [23]. Once inside gill cells, Cd^2+^ induces the synthesis of metallothionein (MT), which binds to Cd^2+^ to form complexes, thereby regulating intracellular cadmium levels and potentially facilitating further uptake [24,25]. Cadmium can also be absorbed through the digestive tract. After fish ingest cadmium-contaminated food (e.g., plankton, benthos), Cd^2+^, which is more readily released in the acidic environment of the digestive tract, enters the body via active transport (e.g., ZIP family zinc transporters) or pinocytosis by intestinal epithelial cells [26,27]. Furthermore, cadmium can be absorbed through osmotic exchange across the body surface, with Cd^2+^ diffusing from water into the fish along its concentration gradient [28].

### 2.2. Cadmium Transport in Fish

Cadmium transport in fish is a dynamic, carrier-mediated process involving multiple routes [29]. After waterborne Cd^2+^ enters the gill tissue, some is adsorbed by the mucus layer but can still penetrate into the circulatory system; another portion is actively transported into gill cells via carriers such as calcium channel proteins [30]. Cadmium absorbed through the digestive tract binds to MT in intestinal epithelial cells before entering the blood. In the bloodstream, most cadmium is bound to MT or albumin, forming complexes that are distributed throughout the body via blood circulation. In the intestine, some Cd^2+^ is excreted in feces, while Cd-MT complexes can be transported directly from the intestinal mucosa to the kidney. Free Cd^2+^ can enter cells via transporters such as divalent metal transporter 1 (DMT1) [31]. The liver is the primary target organ for cadmium accumulation. Cd^2+^ enters hepatocytes via receptor-mediated endocytosis; some is stored after binding to newly synthesized MT, some is excreted in bile, and the remainder enters the kidney via the bloodstream (Figure 1B,C). In the kidney, large amounts of cadmium accumulate in the proximal tubules through glomerular filtration or tubular reabsorption [32,33]. A small amount of cadmium is distributed to muscles and bones via body fluids or interstitial fluid, where it competitively displaces calcium ions in bones and exists as free ions or low-molecular-weight complexes in muscles [34].

### 2.3. Cadmium Accumulation in Fish

The accumulation and distribution of cadmium in fish exhibit significant species- and tissue-specificity [35]. The capacity for cadmium accumulation varies among fish at different trophic levels, generally following the pattern: carnivorous > omnivorous > herbivorous [36,37]. However, unlike mercury (Hg), cadmium does not show significant biomagnification along the food chain; its accumulation is more influenced by exposure routes, ecological niche, and environmental factors [38,39]. Carnivorous fish such as black carp (*Mylopharyngodon piceus*), yellow catfish (*Tachysurus fulvidraco*), and Japanese seabass (Lateolabrax japonicus) often inhabit mid-to-bottom water layers, facing dual exposure from water and sediment. Additionally, the small fish and shrimp they consume tend to have higher cadmium enrichment, resulting in generally higher cadmium loads in these species [40]. Notably, benthic omnivorous fish such as common carp (*Cyprinus carpio*) and crucian carp (*Carassius auratus*) can accumulate cadmium at levels even higher than carnivorous fish in some cases, due to direct contact with sediment and consumption of benthic organisms (e.g., chironomid larvae) [41]. Herbivorous fish such as grass carp (*Ctenopharyngodon idella*) and bluntnose black bream (*Megalobrama amblycephala*) typically inhabit upper water layers and feed mainly on phytoplankton or aquatic plants. Because plants generally have lower cadmium enrichment factors than animal prey, and their waterborne exposure route is relatively simple, herbivorous fish usually exhibit lower cadmium accumulation levels than omnivorous and carnivorous fish [42]. These findings indicate that cadmium accumulation in fish is closely related not only to trophic level but also to ecological niche, feeding habits, and exposure environment. Furthermore, the distribution of cadmium among tissues is highly heterogeneous and is influenced by factors such as exposure route, concentration, and duration. Overall, the liver and kidneys are the primary organs for cadmium accumulation, followed by the gills and intestines, while cadmium levels in muscle are generally low (Table 1). This distribution pattern is closely associated with cadmium’s binding affinity for metallothionein (MT), the metabolic activity of the organs, and cadmium excretion pathways.

**Table 1:** Bioconcentration Patterns in Fish Exposed to Cadmium.

Types		Exposure Method	Exposure Concentration	Exposure Time	Reserve Overview	References
Freshwater fish	*Carassius gibelio*	Waterborne exposure	0.2 mg/L	14 d	Gills > Liver > Intestines > Muscle	[43]
	*Prochilodus lineatus*	Waterborne exposure	10 ug/L	96 h	Kidney > Gills > Liver	[44]
	*Danio rerio*	Waterborne exposure	10 ug/L	21 d	Intestines > Liver	[45]
	*Oreochromismossambicus*	Waterborne exposure	0.5 mg/L	30 d	Liver > Gills > Scales > Muscle	[41]
			1 mg/L	15 d	Kidney > Liver > Gills	[46]
	*Oncorhynchus mykiss*	Waterborne exposure	3.0 ± 07 μg/L	30 d	Kidney > Gills > Liver	[47]
		Dietary exposure	300 μg/g	30 d	Gills > Liver > Kidney	[48]
saltwater fish	*Paralichthys* *olivaceus*	Waterborne exposure	10, 50, 100 μg/L	30 d	Intestines > Gills > Liver > Kidney > Muscle	[49]
	*Sebastes schlegeli*	Dietary exposure	0.5, 5, 25, 125 mg/kg	60 d	Intestines > Kidney ≈ Liver > Gills > Muscle	[50]
	*Solea* *senegalensis*	Waterborne exposure	6.88 μg/L	14 d	Intestines > Liver > Muscle	[51]
		Dietary exposure	0.2 μg/g			

## 3. Upstream Initiation Mechanisms of Cadmium-Induced Apoptosis in Fish

After entering fish cells, Cd^2+^ triggers a series of upstream molecular events through direct or indirect pathways. These events collectively form the early molecular basis for the initiation of apoptosis. This section systematically reviews three core initiating factors—oxidative stress, disruption of calcium homeostasis, and DNA damage—and further analyzes the key signaling hubs that mediate the transmission of these upstream signals to the apoptotic effector machinery.

### 3.1. Central Role of Oxidative Stress

**Figure 2:**
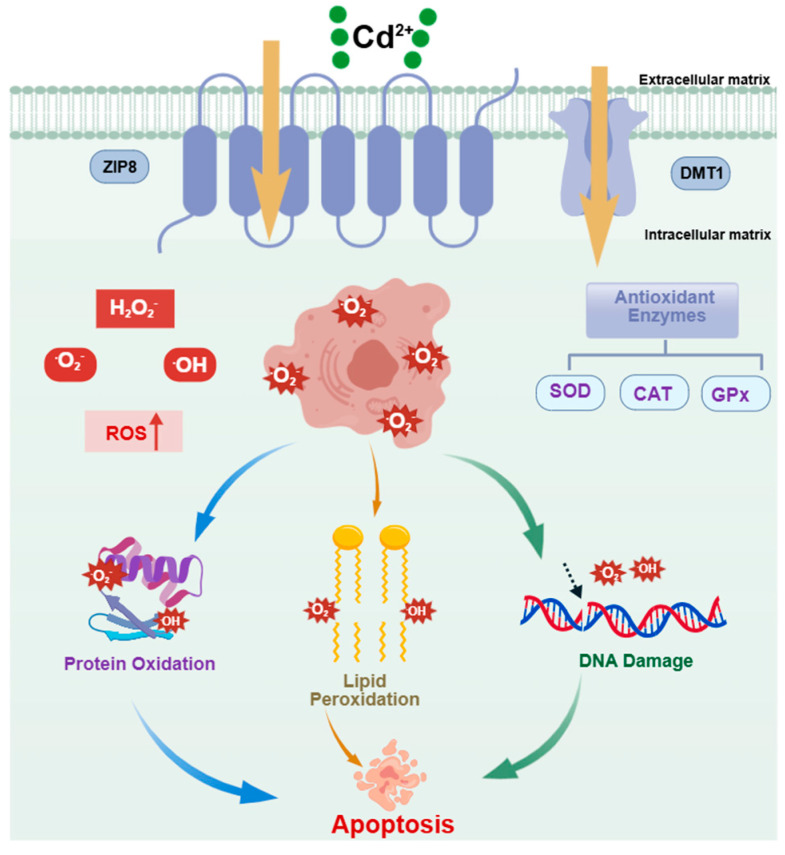
Schematic diagram of cadmium (Cd^2+^)-induced oxidative damage and apoptosis in fish cells.

Oxidative stress (OS) is one of the most important upstream mechanisms by which Cd^2+^ induces apoptosis. As shown in Figure 2, Cd^2+^ exposure induces excessive production of reactive oxygen species (ROS) in fish cells. These highly reactive molecules attack polyunsaturated fatty acids in cell membranes, initiating a lipid peroxidation chain reaction that generates lipid hydroperoxides (LPO) and the end product malondialdehyde (MDA). MDA can cross-link various biological macromolecules, leading to alterations in cell membrane structure and function [51,52,53]. Concurrently, cadmium impairs the function of intracellular antioxidant enzyme systems. Cd^2+^ binds to the sulfhydryl groups of key antioxidant enzymes such as superoxide dismutase (SOD), glutathione peroxidase (GPx), and catalase (CAT), or displaces metal ions in their active centers, forming insoluble thiolates and thereby inhibiting enzyme activity [54,55,56]. Cadmium exposure reduces SOD and GPx activities in the head kidney of common carp, inducing oxidative stress [57]. Li found that cadmium increases ROS and MDA levels in the muscle of Chinese perch by reducing antioxidant enzyme activity, promoting oxidative damage and leading to apoptosis of spleen leukocytes [58]. Notably, the sensitivity to Cd^2+^-induced oxidative stress varies among fish species. For example, at the same exposure concentration, the degree of antioxidant enzyme inhibition is generally higher in benthic fish (e.g., common carp) than in pelagic fish (e.g., tilapia), possibly due to the higher cadmium accumulation load in benthic fish, which are exposed to Cd^2+^ in both water and sediment [59,60].

### 3.2. Calcium Homeostasis Imbalance and Its Dual Regulatory Mechanisms

**Figure 3:**
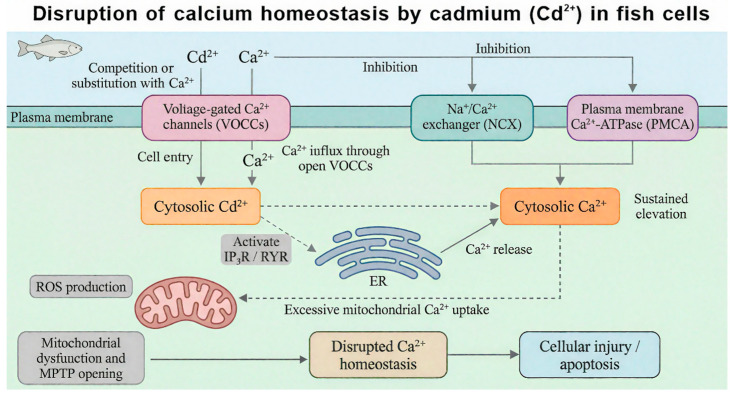
Schematic diagram of calcium homeostasis imbalance induced by Cd^2+^ in fish cells.

Intracellular calcium homeostasis plays a critical role in maintaining cell morphology, function, and signal transduction. As shown in Figure 3, cell damage or stimulation is often accompanied by an increase in intracellular Ca^2+^ concentration, leading to calcium imbalance and further cell injury or death [61,62]. Due to the similar ionic radius and coordination chemistry of Cd^2+^ and Ca^2+^, cadmium interacts with calcium channels in complex ways [63]. On one hand, some studies indicate that Cd^2+^ can mimic Ca^2+^ by binding to Ca^2+^-binding sites on voltage-gated calcium channels (VGCCs) or receptor-gated calcium channels (e.g., NMDA receptors) on the cell membrane, increasing channel open probability and resulting in substantial Ca^2+^ influx [64,65]. On the other hand, Cd^2+^ is also widely regarded as a competitive antagonist of calcium channels, capable of blocking Ca^2+^ entry via VGCCs and TRP channels in various fish models. Which of these dual effects predominates depends on Cd^2+^ concentration, exposure duration, cell type, and channel subtype. Generally, low Cd^2+^ concentrations may mimic Ca^2+^ and promote influx, whereas high concentrations tend to block the channels [66]. Cadmium can also act on intracellular calcium stores such as the endoplasmic reticulum (ER) [67], increasing calcium release while inhibiting calcium reuptake. This leads to a sustained elevation of intracellular calcium concentration, activating calcium-dependent proteases and endonucleases, and causing structural and functional damage to cells. Lee [68] found that Cd^2+^ exposure impairs osmoregulation and ion regulation in fish, activates ER stress in common carp, interferes with normal Ca^2+^ transport, and subsequently induces mitochondrial calcium overload and brain cell apoptosis. Current research on the effects of Cd^2+^ on fish calcium channels has focused mainly on freshwater fish [65,69,70], with relatively few data available for marine fish [71].

### 3.3. Genotoxic Effects of DNA Damage

After entering fish, Cd^2+^ can induce genetic damage through both direct and indirect pathways, disrupting the normal structure and function of DNA [55]. The main manifestations of this genotoxicity include: (i) DNA strand breaks; (ii) base damage and oxidative modifications; (iii) chromosomal aberrations; (iv) DNA-protein crosslinks (DPCs); and (v) inhibition of DNA polymerase activity and DNA replication arrest. On one hand, Cd^2+^ can directly bind to DNA, forming Cd-DNA complexes that disrupt the integrity of the DNA double helix [72]. On the other hand, ROS induced by Cd^2+^ attack DNA molecules, causing oxidative base damage (e.g., 8-OHdG) and deoxyribose backbone breaks [73]. Together, these lesions impair DNA replication and transcription, ultimately leading to gene mutations, cell cycle arrest, and initiation of apoptosis. Multiple studies have confirmed the DNA-damaging effects of Cd^2+^ in fish. Silva showed that exposing striped catfish (*Prochilodus lineatus*) to 1 μg/L and 10 μg/L Cd^2+^ for 96 h resulted in metal accumulation and a significant increase in DNA strand breaks in erythrocytes [44]. Wu reported that exposure of yellow catfish (*Pelteobagrus fulvidraco*) to low concentrations of cadmium caused significant DNA strand breaks in liver tissue, accompanied by elevated levels of DNA damage biomarkers [74]. Gao found that zebrafish (*Danio rerio*) hepatocytes exposed to cadmium-loaded hydroxyapatite nanoparticles (nHAP-Cd) exhibited significant DNA damage and micronucleus formation [75]. Park observed in zebrafish embryos that Cd^2+^ exposure combined with heat stress significantly exacerbated DNA methylation abnormalities and the occurrence of embryonic apoptosis [76].

### 3.4. Signaling Pathway Hubs: Connecting Upstream Signals to Apoptotic Execution Programs

Oxidative stress, calcium imbalance, and DNA damage do not directly activate the downstream apoptotic execution machinery. Instead, they are integrated, amplified, and transmitted to the apoptotic execution pathways (death receptor, mitochondrial, and ER stress pathways) through a series of signal transduction hubs—primarily the MAPK, NF-κB, PI3K/AKT, and Nrf2 pathways. These pathways serve as critical relay stations in Cd^2+^-induced apoptosis in fish cells.

#### 3.4.1. MAPK Signaling Pathway

The mitogen-activated protein kinase (MAPK) family mainly includes three subfamilies: extracellular signal-regulated kinase 1/2 (ERK1/2), c-Jun N-terminal kinase (JNK), and p38 MAPK [77]. Reactive oxygen species (ROS) and calcium (Ca^2+^) signals induced by cadmium (Cd^2+^) activate JNK and p38 MAPK [78,79,80]. Activation of ERK1/2 is concentration-dependent: low concentrations of Cd^2+^ transiently induce ERK1/2-mediated pro-survival signals, while high concentrations inhibit ERK1/2 [81] or, through upstream kinases such as apoptosis signal-regulating kinase 1 (ASK1), switch to activating JNK/p38-dependent pro-apoptotic pathways. In terms of apoptosis regulation, activated JNK and p38 MAPK function through three synergistic mechanisms: (1) phosphorylating downstream transcription factors (e.g., c-Jun, ATF2) to upregulate transcription of pro-apoptotic proteins Bcl-2 interacting mediator of cell death (*Bim*) and p53 upregulated modulator of apoptosis (*PUMA*) and the death ligand Fas ligand (*FasL*); (2) directly phosphorylating Bcl-2 family members, inhibiting the anti-apoptotic functions of Bcl-2 and Bcl-xL while enhancing the pro-apoptotic activities of Bax and Bid; and (3) JNK also enhances the transcriptional activity of p53 through phosphorylation, thereby activating the mitochondrial apoptotic pathway. Evidence from fish studies shows that Cd^2+^ induces mitochondrial apoptosis in common carp (*Cyprinus carpio*) gill cells by activating the JNK-Forkhead box O3a (*FoxO3a*)-PUMA pathway [82]. Zhang found that cadmium induces ROS to upregulate the MAPK pathway (JNK/p38/ERK), phosphorylating transcription factors such as c-Jun, ultimately inducing apoptosis in gill tissue cells of the greenfin horse-faced filefish [83].

#### 3.4.2. NF-κB Signaling Pathway

Nuclear factor kappa B (NF-κB) is a key transcription factor regulating inflammation, immunity, and apoptosis. In its resting state, NF-κB binds to the inhibitory protein IκB and remains inactive in the cytoplasm [84]. Upon cadmium (Cd^2+^) exposure, the induced ROS activate the IκB kinase (IKK) complex, leading to phosphorylation of IκB and its degradation via the ubiquitin-proteasome pathway. This releases NF-κB, which translocates to the nucleus and initiates transcription of target genes [11]. NF-κB plays a dual role in apoptosis regulation depending on the intensity and duration of the stress. Under mild stress, NF-κB primarily exerts pro-survival functions by transcriptionally upregulating anti-apoptotic proteins (e.g., *Bcl-xL*, *Bcl-2*, *c-IAP1/2*, *FLIP*) to inhibit caspase activation. Under sustained or strong stress conditions, NF-κB can also upregulate pro-apoptotic factors (e.g., *Fas*, *FasL*, *Bax*), particularly in coordination with pathways such as JNK, shifting toward promoting apoptosis [85,86,87]. Studies in fish have shown that Cd^2+^ exposure significantly induces phosphorylation and degradation of IκBα in monocytes/macrophages of the spotted snakehead (Channa punctatus), leading to nuclear translocation of the NF-κB p65 subunit and upregulation of pro-inflammatory factors such as TNF-α and IL-1β [86]. However, whether NF-κB plays a protective or pro-apoptotic role in Cd^2+^-induced apoptosis remains controversial, likely depending on cell type, exposure dose, and exposure duration [88,89].

#### 3.4.3. PI3K/AKT Signaling Pathway

The phosphatidylinositol 3-kinase (PI3K)/AKT pathway is a central signaling pathway for cell survival, proliferation, and metabolism [90]. Under physiological conditions, PI3K is activated by growth factor receptors or stress signals, catalyzing the conversion of PIP2 to PIP3, which in turn recruits and activates AKT [91]. Activated AKT exerts anti-apoptotic effects by phosphorylating various substrates: (1) phosphorylating Bad, promoting its dissociation from Bcl-xL; (2) phosphorylating Bax, inhibiting its translocation to mitochondria; (3) phosphorylating caspase-9, inhibiting its activity; (4) activating NF-κB (via IKK); and (5) inhibiting FoxO transcription factors, reducing the expression of pro-apoptotic genes [92,93,94,95]. However, under cadmium (Cd^2+^) exposure, this pathway is often inhibited. The mechanism may involve Cd^2+^-induced oxidative stress increasing the activity of PTEN (a PIP3 phosphatase), or Cd^2+^ directly inhibiting the catalytic activity of PI3K. Reduced AKT activity relieves its inhibition of Bad, Bax, and caspase-9, thereby promoting mitochondrial apoptosis [96]. Currently, research on the regulation of the PI3K/AKT pathway by Cd^2+^ in fish remains limited. Luan [97] found that Cd^2+^ upregulates *FKBP5* protein expression by modulating *miR-9-5p*, thereby activating the PI3K/AKT signaling pathway and inducing apoptosis in carp lymphocytes, suggesting that this pathway may be involved in Cd^2+^-induced apoptosis in fish. However, the underlying mechanisms require further investigation.

#### 3.4.4. Nrf2 Antioxidant Pathway

Nuclear factor erythroid 2-related factor 2 (Nrf2) is a master regulator of cellular antioxidant defense [98]. Under physiological conditions, Nrf2 binds to Keap1 in the cytoplasm and is ubiquitinated and degraded. Under cadmium (Cd^2+^) exposure, Nrf2 can be activated through the following mechanisms: (1) Cd^2+^ modifies the sulfhydryl groups of critical cysteine residues in Keap1, altering its conformation and promoting Nrf2 dissociation; (2) Cd^2+^ induces p62 expression, and p62 competitively binds to Keap1, releasing Nrf2. Free Nrf2 translocates to the nucleus, forms heterodimers with small Maf proteins, binds to the antioxidant response element (ARE), and initiates transcription of downstream genes (e.g., heme oxygenase-1 (HO-1), NAD(P)H quinone dehydrogenase 1 (NQO1), glutamate-cysteine ligase catalytic subunit (GCLC), glutamate-cysteine ligase modifier subunit (GCLM), superoxide dismutase (SOD), catalase (CAT)). Activation of this pathway is a compensatory protective response to Cd^2+^-induced oxidative stress, enhancing the cell’s ability to clear ROS. However, when Cd^2+^ concentrations are too high or exposure is prolonged, Nrf2 nuclear translocation and transcriptional activity may be inhibited, leading to collapse of the antioxidant defense system and exacerbation of oxidative damage and apoptosis [99,100,101]. In fish studies, Li [58] reported that Cd^2+^ exposure inhibits the *miR-216a*-mediated Nrf2 pathway in the muscle of Chinese perch (Siniperca chuatsi), reducing *HO-1* and *GCLC* expression and exacerbating oxidative stress and apoptosis. Conversely, exogenous antioxidants (e.g., selenium, vitamin C) can activate the Nrf2 pathway and alleviate Cd^2+^ toxicity [80]. These findings indicate that Nrf2 is a key regulatory node in Cd^2+^ toxicity and a potential target for protective intervention.

#### 3.4.5. Interaction Network of Signaling Pathways

**Figure 4:**
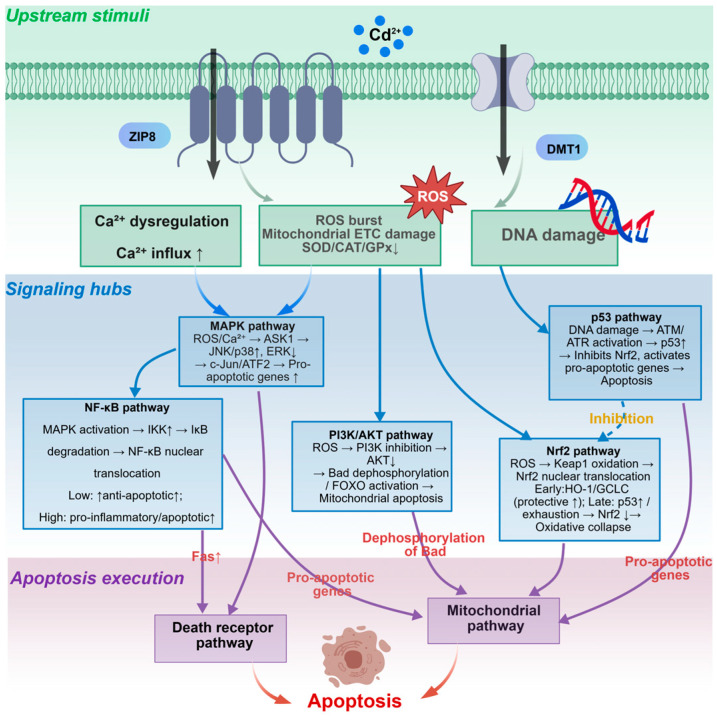
Upstream signal integration and interaction network in cadmium (Cd^2+^)-induced apoptosis in fish cells.

The four signaling pathways described above do not operate independently; instead, they form a complex interaction network through multiple molecular mechanisms. As shown in Figure 4, Cd^2+^-induced ROS simultaneously activate the JNK/p38 MAPK, NF-κB, and Nrf2 pathways. JNK can inhibit PI3K/AKT and enhance NF-κB activity. There is competitive antagonism between NF-κB and Nrf2 for transcriptional coactivators (CBP/p300). AKT positively regulates NF-κB. Additionally, p53 activated by DNA damage upregulates *Bax* and inhibits Nrf2, serving as another integration node. This network regulation confers threshold dependence on Cd^2+^-induced apoptotic signals: under low-intensity stimulation, the Nrf2 protective response and the NF-κB pro-survival branch dominate, and cells survive; under high-intensity stimulation, JNK/p38 are strongly activated, PI3K/AKT is inhibited, and NF-κB shifts toward pro-apoptosis. The superimposed signals exceed the threshold, irreversibly initiating the caspase cascade [63,102,103,104,105]. However, research in this area in fish still has notable limitations: most studies have examined only one or two key proteins in a single pathway, lacking dynamic analysis of multiple pathways; species differences, tissue specificity, and adaptive changes under long-term exposure to environmentally relevant concentrations remain unexplored; and existing conclusions are largely based on correlative analyses without validation. Future research should combine multiplex detection, phosphoproteomics, genetic intervention, and environmentally relevant exposure protocols to systematically dissect the signaling network of Cd^2+^ toxicity in fish.

## 4. Downstream Execution Pathways of Cadmium-Induced Apoptosis in Fish

The high toxicity of cadmium in fish cells is reflected in its ability to coordinately activate multiple apoptotic pathways, including the extrinsic death receptor pathway, the intrinsic mitochondrial pathway, and the ER stress pathway. This section sequentially describes the activation mechanisms of each pathway and then focuses on analyzing the crosstalk among them.

### 4.1. Death Receptor Pathway (Extrinsic Pathway)

**Figure 5:**
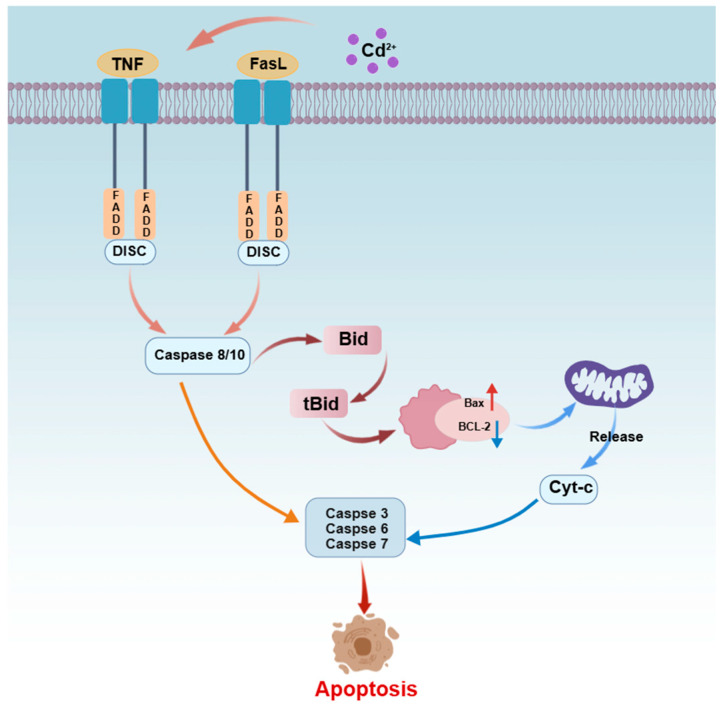
Schematic diagram of the cadmium (Cd^2+^)-mediated death receptor pathway.

The death receptor pathway, also known as the extrinsic apoptotic pathway, is mediated by transmembrane death receptors (e.g., Fas, TNFR1) that transduce extracellular apoptotic signals into the cell to initiate apoptosis [65,106]. As shown in Figure 5, upon Cd^2+^ exposure in fish cells, activation of death receptors recruits the adaptor protein FADD, which binds to procaspase-8/10 to form the death-inducing signaling complex (DISC). This complex catalyzes the activation of the initiator caspases, caspase-8/10. Activated caspase-8/10 then activate effector caspases (e.g., caspase-3, -6, and -7) through two pathways: directly cleaving procaspase-3, or cleaving the Bid protein (producing tBid) to transmit the signal to the mitochondrial pathway, thereby amplifying the signal [107,108]. Fish studies have shown that Cd^2+^ can enhance the activity of the death receptor pathway by transcriptionally upregulating its key components. For example, Zhang [57] reported that Cd^2+^ exposure significantly increased the mRNA and protein levels of *Fas*, *FADD*, *caspase-8*, and *caspase-3* in head kidney lymphocytes of common carp, accompanied by increased apoptosis. Jiaxin observed that Cd^2+^ stress induced upregulation of mRNA expression of apoptosis-related genes including tumor necrosis factor alpha (*TNF-α*), TNF receptor 1 (*TNFR1*), *Fas*, *FasL*, and *Bax* in carp neutrophils, leading to cell apoptosis via the death receptor pathway [109].

### 4.2. Mitochondrial Pathway (Intrinsic Pathway)

**Figure 6:**
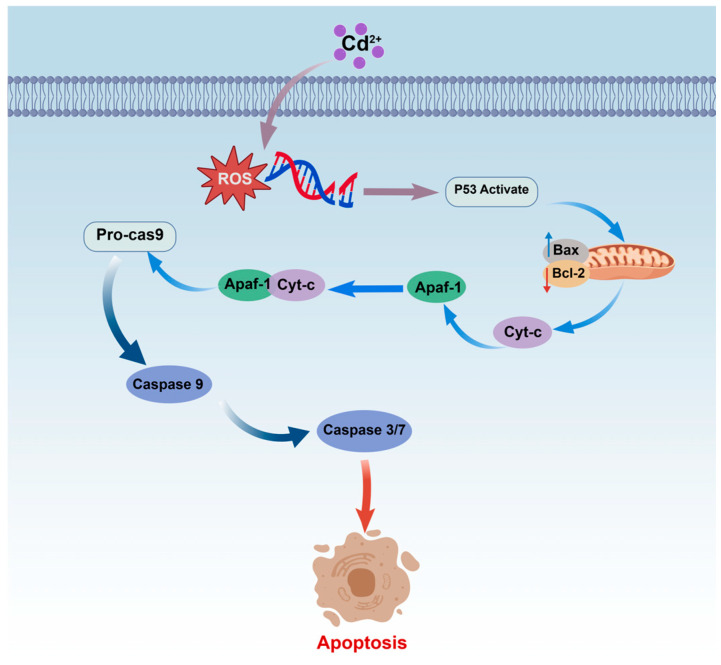
Schematic diagram of the cadmium (Cd^2+^)-mediated mitochondrial apoptotic pathway.

The mitochondrial pathway (Figure 6) plays a central role in Cd^2+^-induced apoptosis, with mitochondria acting as key signal integrators that mediate multilayered death signals [110,111]. In upstream regulation, the tumor suppressor protein p53 responds to stresses such as DNA damage by transcriptionally upregulating the pro-apoptotic protein *Bax* and downregulating the anti-apoptotic protein *Bcl-2*, thereby increasing the Bax/Bcl-2 ratio. This increased ratio promotes Bax oligomerization on the outer mitochondrial membrane, forming pores and inducing mitochondrial outer membrane permeabilization (MOMP). Consequently, cytochrome c (Cyt-C) and apoptosis-inducing factor (AIF) are released from the mitochondrial intermembrane space into the cytoplasm. Released Cyt-C assembles with apoptotic protease activating factor 1 (Apaf-1) and procaspase-9 to form the apoptosome, which activates procaspase-9 to produce initiator caspase-9. Caspase-9 then cleaves and activates effector caspase-3, executing the classical caspase-dependent apoptotic program [82,108,112,113]. Concurrently, Cd^2+^ exposure can also damage mitochondrial DNA (mtDNA), triggering AIF translocation to the nucleus, where it mediates chromatin condensation and DNA fragmentation, thereby initiating a caspase-independent apoptotic pathway [114,115]. Studies in fish have shown that Cd^2+^ exposure induces kidney injury in common carp through impaired mitochondrial energy metabolism and mitochondria-dependent apoptosis, accompanied by significant activation of caspase-9 and caspase-3 [111]. Zheng demonstrated in common carp that Cd^2+^ induces reactive oxygen species (ROS) generation, leading to mitochondrial swelling and cytochrome c release, and activates *p53* gene expression, ultimately causing apoptosis via the mitochondrial apoptotic pathway [116].

### 4.3. Endoplasmic Reticulum Stress-Mediated Apoptotic Pathway

**Figure 7:**
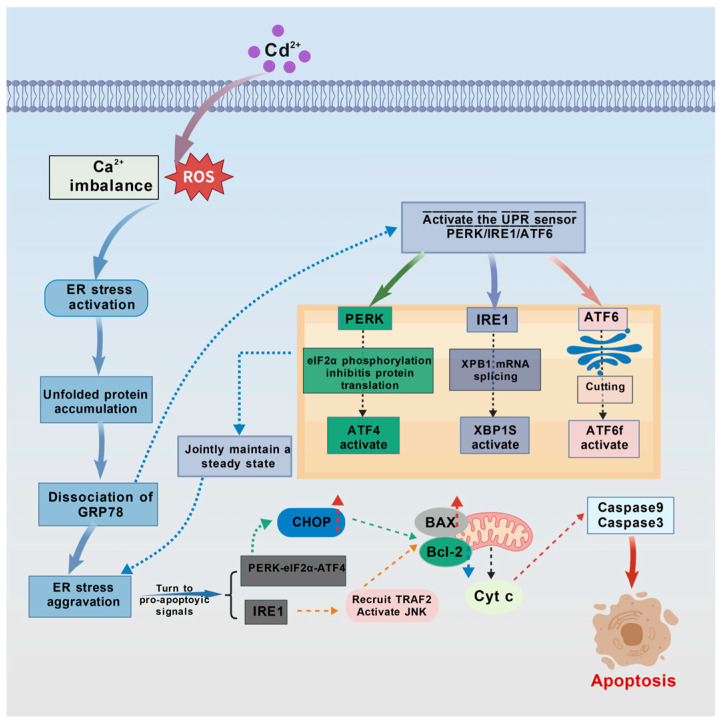
Schematic diagram of the cadmium (Cd^2+^)-mediated endoplasmic reticulum apoptotic pathway.

The endoplasmic reticulum (ER) stress-mediated apoptotic pathway is an important component of the intrinsic apoptotic pathway (Figure 7). Cadmium induces ER stress by disrupting protein folding and calcium homeostasis within the ER lumen. Under normal physiological conditions, ER stress initiates the unfolded protein response (UPR), which restores cellular homeostasis through three sensing pathways: PERK-eIF2α, IRE1-XBP1, and ATF6. However, sustained or excessive ER stress overwhelms the adaptive capacity of the UPR, shifting it toward activating an apoptotic signaling cascade that leads to cell death [117,118,119]. It is important to note that there is close crosstalk between the ER stress pathway and the mitochondrial pathway, primarily mediated by Ca^2+^ signaling and BH3-only proteins. In the following subsections, each branch pathway is described with an emphasis on these interaction nodes.

#### 4.3.1. PERK-eIF2α-Mediated Apoptosis

In ER stress, PERK, which is localized to the ER membrane, is a key sensor of the unfolded protein response. Under non-stressed conditions, PERK is bound to the chaperone BiP/GRP78 and remains inactive [120]. Upon exposure of fish to Cd^2+^, the accumulation of misfolded proteins in the ER lumen competitively binds BiP/GRP78, promoting PERK dissociation, oligomerization, and autophosphorylation, thereby activating it. Activated PERK phosphorylates eukaryotic translation initiation factor 2α (eIF2α). Initially, phosphorylated eIF2α reduces the global protein synthesis rate, alleviating the ER protein folding load and exerting a cytoprotective effect. However, under sustained or severe ER stress, p-eIF2α promotes the translation of the transcription factor ATF4, which upregulates the pro-apoptotic protein CHOP/GADD153, initiating apoptosis [66,121,122,123]. CHOP can indirectly induce mitochondrial MOMP by downregulating *Bcl-2* and upregulating BH3-only proteins such as *Bim* and *PUMA*, thereby transmitting signals from ER stress to the mitochondrial apoptotic pathway [124]. Fish studies have confirmed that cadmium exposure induces ER stress: increased dietary cadmium levels in juvenile black seabream trigger ER stress, leading to significantly elevated Grp78 and ATF6 levels [125]. Exposure of common carp to cadmium increases Grp78 expression in the gills and elevates PERK, ATF4, and ATF6 levels, leading to cell apoptosis [126].

#### 4.3.2. IRE1/XBP1-Mediated Apoptosis

IRE1 is the second key sensor molecule and transmembrane kinase on the ER membrane, and its activation mechanism is similar to that of PERK [127]: accumulation of misfolded proteins promotes dissociation of IRE1 from the BiP/GRP78 inhibitory complex, followed by oligomerization and autophosphorylation, leading to its activation. Activated IRE1 promotes apoptosis mainly through two pathways. First, it recruits the cytoplasmic adaptor protein TNF receptor-associated factor 2 (TRAF2), which on one hand activates c-Jun N-terminal kinase (JNK). JNK inactivates anti-apoptotic proteins (e.g., Bcl-2, Bcl-xL) and activates pro-apoptotic proteins (e.g., Bim) through phosphorylation, thereby initiating the mitochondrial apoptotic pathway. On the other hand, TRAF2 also activates caspase-12, triggering a downstream caspase cascade. Second, activated IRE1 uses its endonuclease activity to cleave X-box binding protein 1 (XBP1) mRNA, producing the spliced and activated form spliced XBP1 (sXBP1). sXBP1 upregulates the transcription of UPR target genes, including both protective chaperones and the pro-apoptotic factor CHOP, which together promote apoptosis under severe stress [127,128,129].

In mammals, the IRE1-TRAF2 complex can activate caspase-12, which serves as an ER stress-specific initiator caspase in the apoptotic process. However, in teleost fish, a homolog of caspase-12 has not yet been formally identified, and its function has not been validated. Most conclusions regarding caspase-12 in current fish studies are extrapolated from mammalian data. Future research should use experimental approaches such as gene cloning and knockdown to determine whether fish possess an ER stress-specific caspase functionally equivalent to mammalian caspase-12 [130,131].

#### 4.3.3. ATF6-Mediated Apoptosis

ATF6 is a type I transmembrane protein on the ER membrane. Its N-terminal intracellular region contains a b-ZIP DNA transcription activation domain and a nuclear localization signal. Under non-stressed conditions, ATF6 is distributed on the ER membrane as a zymogen [132]. Under ER stress, ATF6 is transported to the Golgi apparatus via vesicles. In the Golgi, it is cleaved and activated by S1P and S2P proteases. It then translocates to the nucleus, guided by its nuclear localization signal, where it induces the transcription of ER stress genes, including *CHOP*/*GADD153*, thereby promoting apoptosis [133].

#### 4.3.4. Calcium Homeostasis Dysregulation and ER–Mitochondria Crosstalk

Under normal cellular conditions, the ER maintains calcium homeostasis by releasing Ca^2+^ from its lumen into the cytoplasm through RyR and IP3R channels and pumping cytoplasmic Ca^2+^ back into the ER lumen via calcium pumps. Cadmium ions (Cd^2+^) can displace Ca^2+^ in the ER lumen, not only directly disrupting calcium homeostasis but also occupying the Ca^2+^-binding sites of calcium-dependent chaperones such as calnexin and calreticulin. This causes these chaperones to lose their normal lectin-like structure and glycoprotein folding function, leading to the accumulation of misfolded proteins in the ER lumen—a key molecular basis for Cd^2+^-triggered ER stress. Under ER stress, dysregulated Ca^2+^ efflux causes the ER to release excessive Ca^2+^ into the cytoplasm. The persistently elevated cytoplasmic Ca^2+^ is taken up by mitochondria via the mitochondrial calcium uniporter (MCU), causing mitochondrial Ca^2+^ overload. This triggers MOMP through mechanisms such as inducing the opening of the mitochondrial permeability transition pore (mPTP), promoting Bax translocation and oligomerization, and increasing the efficiency of cytochrome c release. Ultimately, this abnormal Ca^2+^ signaling promotes apoptosis through two pathways: (1) disrupting mitochondrial function and modulating the activity of Bcl-2 family proteins, thereby activating the intrinsic mitochondrial apoptotic pathway; and (2) activating the calcium-dependent cysteine protease calpain, which initiates the caspase cascade by cleaving and activating procaspase [66,81,134,135].

### 4.4. Crosstalk Network Among Apoptotic Pathways

**Figure 8:**
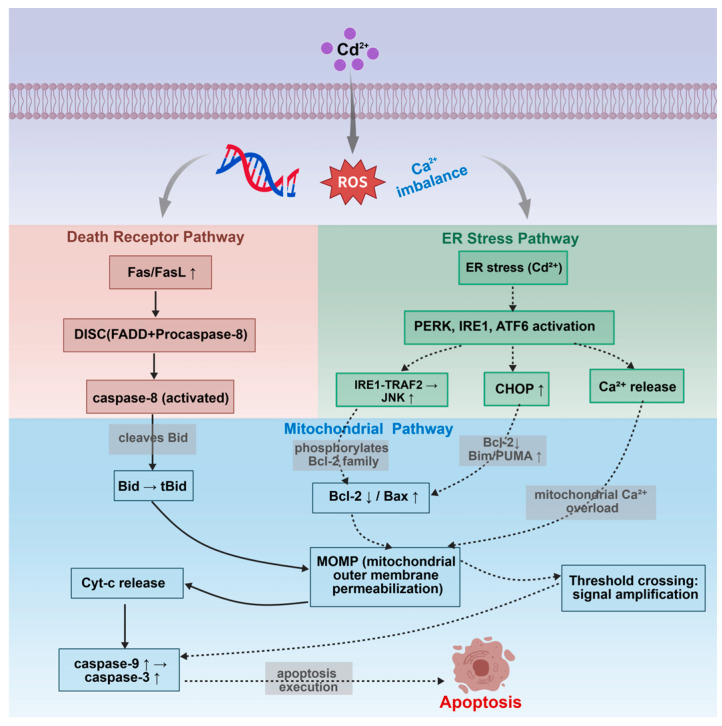
Interaction network of the three major pathways in cadmium (Cd^2+^)-induced apoptosis in fish cells.

Cd^2+^-induced apoptosis in fish cells is not executed by a single pathway independently, but rather through a complex interaction network formed by the three major apoptotic pathways via multiple molecular nodes (Figure 8). Specifically, activated caspase-8 in the death receptor pathway cleaves Bid to produce tBid, which translocates to mitochondria and induces mitochondrial outer membrane permeabilization (MOMP), thereby achieving cascade amplification of death receptor signals to the mitochondrial pathway. Concurrently, the ER stress pathway interacts with the mitochondrial pathway through two routes: on one hand, Ca^2+^ released during ER stress is taken up by mitochondria, causing mitochondrial Ca^2+^ overload and triggering MOMP; on the other hand, JNK activated by the IRE1-TRAF2 complex phosphorylates Bcl-2 family members, inhibiting anti-apoptotic proteins and activating pro-apoptotic proteins, also promoting mitochondrial MOMP. Furthermore, all three ER stress branches (PERK, IRE1, and ATF6) can upregulate the transcription factor CHOP, which indirectly activates the mitochondrial apoptotic pathway by downregulating *Bcl-2* and upregulating *Bim* and *PUMA* [63,79,136]. These interactive mechanisms allow low-intensity signals from a single pathway to be amplified through the network, ultimately exceeding the apoptotic threshold. This explains why cadmium, as a multi-target toxicant, typically induces apoptosis more efficiently than stimuli that specifically activate a single pathway.

## 5. Conclusions

In summary, cadmium (Cd^2+^), as a typical heavy metal pollutant in aquatic environments, enters fish through the gills, digestive tract, and body surface, and accumulates mainly in the liver and kidneys. It initiates apoptotic signals through oxidative stress, calcium imbalance, and DNA damage, which are then executed coordinately by the death receptor, mitochondrial, and ER stress pathways. The mitochondrial pathway plays a central role, and the three pathways form a complex interaction network through nodes such as Bid, Ca^2+^, JNK, and CHOP, jointly amplifying the apoptotic effect. Cadmium-induced apoptosis exhibits clear dose dependence: long-term exposure to low concentrations (<10 μg/L) primarily activates protective responses; moderate concentrations (10–100 μg/L) lead to detectable caspase-3 activation and decreased mitochondrial membrane potential; high concentrations (>100 μg/L) strongly activate all three pathways and exceed the apoptotic threshold. Nutritional protective agents (e.g., selenium, vitamins C/E, and taurine) effectively alleviate apoptosis through mechanisms such as direct antioxidant activity, Cd^2+^ chelation, activation of the Nrf2 pathway, and inhibition of the caspase cascade. However, the efficacy of different protective agents and their combined application strategies requires further evaluation. Current research still has limitations, including unclear dynamic pathway interactions, a lack of studies on low-dose chronic exposure and intergenerational effects, neglect of species differences, insufficient translational application of protective agents, and the unresolved identity of fish-specific apoptotic molecules (e.g., caspase-12 homologs). Future research should focus on constructing multidimensional dose–time–response maps, elucidating the activation thresholds and interaction contributions of apoptotic pathways, and developing composite protective strategies based on Nrf2 activators, metal chelators, and antioxidants. This will promote the translation of mechanistic research into ecological risk assessment and pollution control in aquaculture.

## Data Availability

No new data were created or analyzed in this study. Data sharing is not applicable to this article.
